# Incretin-based cardiovascular protection beyond diabetes: evidence, mechanisms, and therapeutic frontiers from GLP-1 receptor agonists to multi-agonist therapy

**DOI:** 10.3389/fendo.2026.1870649

**Published:** 2026-07-02

**Authors:** Libin Qiu, Jinpeng Liu, Nana Hu

**Affiliations:** 1 Structural Cardiology Department, Weifang People’s Hospital (The First Affiliated Hospital of Shandong Second Medical University), Weifang, Shandong, China; 2 Cardiology Department 2, Weifang People’s Hospital (The First Affiliated Hospital of Shandong Second Medical University), Weifang, Shandong, China; 3 Cardiology Department 1, Weifang People’s Hospital (The First Affiliated Hospital of Shandong Second Medical University), Weifang, Shandong, China

**Keywords:** cardiovascular outcomes, GLP-1 receptor agonists, incretin therapy, MACE, multi-agonist therapy

## Abstract

Incretin-based therapy has moved from glycaemic control into the centre of cardiometabolic medicine. Glucagon-like peptide-1 receptor agonists (GLP-1 RAs) consistently reduce major adverse cardiovascular events (MACE) in patients with type 2 diabetes (T2DM) and established atherosclerotic cardiovascular disease (ASCVD), while semaglutide 2.4 mg has extended the evidence base to people with overweight or obesity and established cardiovascular disease in the absence of diabetes. Dedicated trials have further expanded the clinical landscape to chronic kidney disease (CKD), obesity-related heart failure with preserved ejection fraction (HFpEF), and symptomatic peripheral artery disease (PAD). Concurrently, dual and triple incretin-based agonists, oral peptide and non-peptide formulations, and combination strategies with sodium-glucose cotransporter-2 (SGLT2) inhibitors and non-steroidal mineralocorticoid receptor antagonists are reshaping treatment algorithms. This critical review synthesizes cardiovascular outcome trials (CVOTs), mechanistic studies, meta-analyses, guideline recommendations, and regulatory developments available up to 30 April 2026. The central conclusion is that incretin-based cardiovascular protection is biologically plausible and clinically reproducible. Critically, this benefit is now independent of diabetes as the defining therapeutic context—a paradigm shift with broad implications for cardiovascular practice. Nevertheless, the magnitude of benefit varies considerably by molecule, dose, population, comparator, and endpoint hierarchy; mediation analyses do not precisely quantify direct cardioprotection; and cardiovascular outcome evidence for newer multi-agonists and oral non-peptide GLP-1 RAs remains incomplete. Interpretation therefore requires a balanced evidence framework: GLP-1 RAs with proven outcome benefit should be prioritized in patients with ASCVD (e.g., LEADER, SUSTAIN-6, HARMONY Outcomes), obesity with established CVD (SELECT), CKD (FLOW), or obesity-related HFpEF (STEP-HFpEF) when supported by trial data and regulatory indications, whereas next-generation agents should be evaluated according to completed CVOTs rather than weight-loss efficacy alone. Remaining research priorities include defining weight-independent mechanisms, optimizing combination therapy, identifying biomarker-defined responders, preserving lean mass during high-efficacy weight loss, and ensuring equitable global access.

## Graphical Abstract

**Figure:**
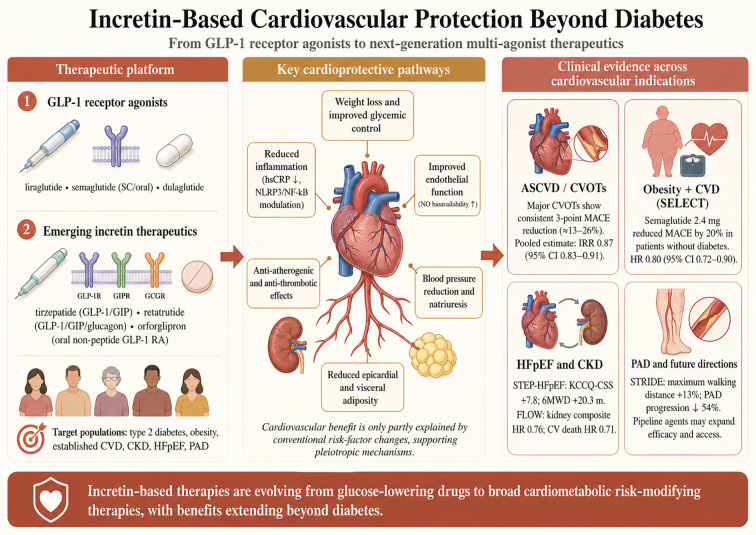
The graphical abstract summarizes the conceptual framework of incretin-based cardiovascular protection beyond diabetes. GLP-1 receptor agonists and next-generation incretin therapeutics, including dual and triple receptor agonists and oral non-peptide agents, are positioned as an expanding therapeutic platform for patients with type 2 diabetes, obesity, established cardiovascular disease, chronic kidney disease, heart failure with preserved ejection fraction, and peripheral arterial disease. Their cardiovascular benefits are mediated through both conventional cardiometabolic risk-factor modification and pleiotropic mechanisms, including attenuation of systemic and vascular inflammation, improved endothelial nitric oxide bioavailability, anti-atherogenic and anti-thrombotic effects, natriuresis and blood pressure reduction, and reduction of epicardial and visceral adiposity. The clinical evidence base spans major cardiovascular outcome trials in atherosclerotic cardiovascular disease, SELECT in obesity-associated cardiovascular disease without diabetes, STEP-HFpEF and FLOW in heart failure and cardiorenal disease, and STRIDE in peripheral arterial disease. Collectively, these data support the evolving role of incretin-based therapies from glucose-lowering drugs to broad cardiometabolic risk-modifying interventions, while underscoring the need for mechanistic clarification, precision patient selection, and equitable implementation.

## 1 Introduction

The cardiovascular interpretation of incretin-based therapy has changed fundamentally over the past decade. Initially developed as glucose-lowering therapy for type 2 diabetes mellitus (T2DM), GLP-1 RAs are now supported by randomized evidence showing reductions in major adverse cardiovascular events (MACE) in selected patients with T2DM and high cardiovascular risk (1–5). Semaglutide outcome trials have extended the evidence base to obesity with established cardiovascular disease, chronic kidney disease, obesity-related HFpEF and symptomatic peripheral artery disease (6–9). The field has therefore shifted from a diabetes-centred question - whether these agents are safe for the heart - to a cardiovascular question: which patient, which molecule, which dose, which comparator and which outcome should guide treatment selection?

This shift was not inevitable. Early incretin trials were shaped by regulatory cardiovascular-safety requirements after concerns regarding glucose-lowering therapies and cardiovascular risk (10, 11). Neutral results with lixisenatide and once-weekly exenatide initially suggested that cardiovascular efficacy might not be a universal property of all incretin agents (12, 13). Subsequent superiority trials with liraglutide, subcutaneous semaglutide, albiglutide, dulaglutide and efpeglenatide established that sustained GLP-1 receptor activation can reduce atherosclerotic events in populations with T2DM and high cardiovascular risk (1–3, 14, 15). More recently, SELECT demonstrated a statistically robust reduction in MACE with semaglutide 2.4 mg in adults with overweight or obesity and established cardiovascular disease without diabetes, decoupling cardiovascular benefit from glucose lowering as the primary explanatory framework (6).

The biological rationale for this expanded role is increasingly coherent. GLP-1 receptors and incretin-related signalling networks are present in tissues relevant to cardiovascular and kidney disease, including vascular endothelium, immune cells, platelets, kidney tubular pathways, central appetite-regulating circuits and, in some experimental systems, cardiomyocytes (16–19). GLP-1 receptor activation engages cyclic AMP-dependent signalling, protein kinase A, EPAC pathways and downstream anti-inflammatory and vasodilatory programmes (16–20). In parallel, clinically important reductions in body weight, visceral adiposity, blood pressure, albuminuria, glycaemia and inflammatory biomarkers create a multifactorial cardioprotective phenotype (6, 7, 21, 22). The key scientific challenge is not whether these factors matter, but how much of the clinical effect is mediated by conventional risk-factor modification and how much reflects tissue-level biology not captured by routine biomarkers.

Several limitations of the current evidence base require explicit consideration. Cardiovascular outcome trials differ substantially in background therapy, event rates, duration, enrolled risk phenotype, statistical hierarchy and comparator choice (4, 5, 23). Semaglutide outcome data cannot be automatically generalized to all GLP-1 RAs, and weight loss efficacy cannot be treated as a substitute for adjudicated cardiovascular outcomes (5–7, 23). The active-comparator design of SURPASS-CVOT provides a stringent test of tirzepatide versus dulaglutide and should be interpreted as noninferiority for MACE rather than established superiority (24). Similarly, oral non-peptide GLP-1 RAs such as orforglipron may transform access and adherence but currently require dedicated cardiovascular outcome data before they can be positioned as cardiovascular risk-reduction therapies (25, 26).

This review provides an integrated synthesis of the clinical evidence, mechanistic rationale, safety considerations, implementation challenges, and unresolved questions surrounding incretin-based cardiovascular protection. It emphasizes trial hierarchy, biological plausibility, safety, implementation, and unresolved questions, with the explicit aim of aligning scientific enthusiasm with evidentiary discipline.

## 2 Methods and evidence framework

This is a critical narrative review with structured evidence mapping, not a PRISMA-style systematic review. PubMed/MEDLINE, Embase, ClinicalTrials.gov, major cardiovascular and diabetes society documents, FDA and WHO regulatory materials, and major congress reports were reviewed for studies available up to 30 April 2026. Search concepts included GLP-1 receptor agonist, glucagon-like peptide-1, incretin, semaglutide, liraglutide, dulaglutide, exenatide, lixisenatide, albiglutide, efpeglenatide, tirzepatide, retatrutide, orforglipron, cardiovascular outcomes, MACE, heart failure with preserved ejection fraction, chronic kidney disease, peripheral artery disease, obesity, safety and access. Because this article is a critical narrative review rather than a formal systematic review, no PRISMA flow diagram, quantitative risk-of-bias assessment, or protocol registration was performed.

Priority was assigned to randomized cardiovascular outcome trials, dedicated kidney or heart-failure trials, prespecified analyses, high-quality meta-analyses, mechanistic studies with causal experimental designs, and official regulatory or guideline documents. Observational studies were considered supportive only when randomized evidence was absent or when addressing implementation, persistence, prescribing disparities, or rare safety outcomes. For each topic, the interpretation distinguishes between outcome-proven evidence, biomarker or functional evidence, mechanistic plausibility and hypothesis-generating data.

The review uses three interpretive rules. First, event-driven CVOTs are considered the highest level of evidence for cardiovascular risk reduction (4, 5). Second, secondary or exploratory endpoints are interpreted in the context of hierarchical testing and multiplicity, a point particularly relevant to active-comparator trials such as SURPASS-CVOT (24). Third, mediation analyses are treated as tools for hypothesis generation rather than precise partitioning of causal mechanisms (27). These rules are essential because the clinical enthusiasm surrounding incretin therapy can otherwise lead to overextension of valid trial findings into populations, molecules or endpoints that have not yet been directly tested.

## 3 Cardiovascular outcome evidence in type 2 diabetes

### 3.1 From safety demonstration to cardiovascular efficacy

**Figure 1:**
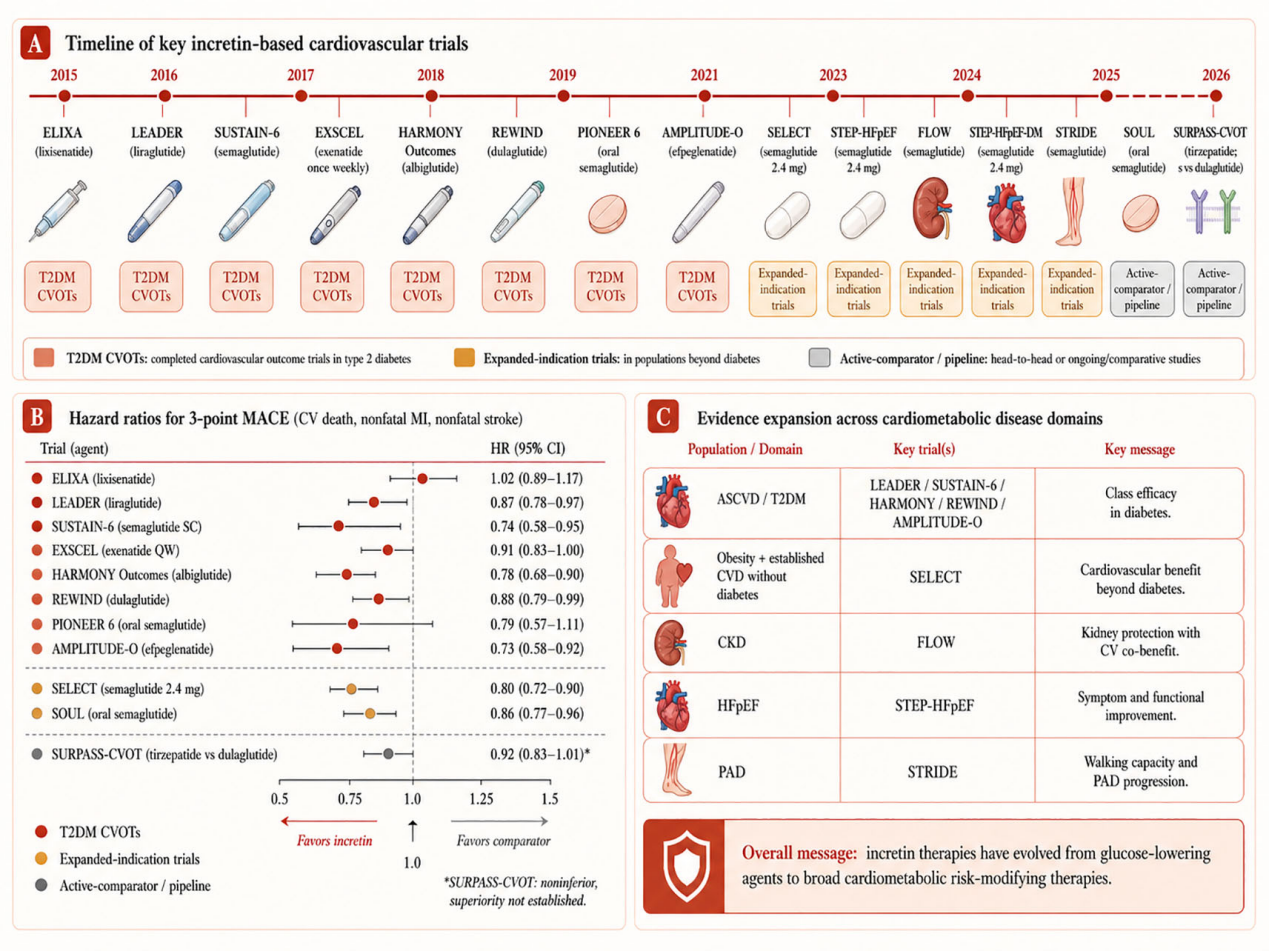
Evolution and outcome landscape of incretin-based cardiovascular trials. This image summarizes the temporal development and outcome evidence of incretin-based cardiovascular and cardiorenal trials. **(A)** Shows the progression from early regulatory cardiovascular outcome trials in type 2 diabetes mellitus to expanded-indication studies in obesity-associated cardiovascular disease, chronic kidney disease, heart failure with preserved ejection fraction, peripheral arterial disease, and active-comparator or next-generation incretin trials. **(B)** Displays representative hazard ratios for 3-point major adverse cardiovascular events across major GLP-1 receptor agonist and incretin-based trials, highlighting the overall consistency of benefit among long-acting GLP-1 receptor agonists while emphasizing neutral or non-superiority findings in selected trials. **(C)** Illustrates the expansion of evidence across cardiometabolic disease domains, including ASCVD with diabetes, obesity with established cardiovascular disease without diabetes, CKD, HFpEF, and PAD. Cross-trial comparisons should be interpreted cautiously because of differences in trial population, baseline cardiovascular risk, comparator, drug exposure, follow-up duration, background therapy, and endpoint definitions. ASCVD, atherosclerotic cardiovascular disease; CKD, chronic kidney disease; CV, cardiovascular; CVOT, cardiovascular outcome trial; HFpEF, heart failure with preserved ejection fraction; HR, hazard ratio; MACE, major adverse cardiovascular events; MI, myocardial infarction; PAD, peripheral arterial disease; T2DM, type 2 diabetes mellitus.

The earliest GLP-1 RA CVOTs established cardiovascular safety but also revealed heterogeneity within the class. ELIXA enrolled patients with T2DM shortly after acute coronary syndrome and demonstrated that lixisenatide was cardiovascularly safe, with a neutral effect on 3-point MACE (12). Because lixisenatide is a short-acting GLP-1 RA with predominantly postprandial effects and limited sustained systemic receptor occupancy, ELIXA should not be read as evidence against the broader class; rather, it highlighted that pharmacology and exposure matter. EXSCEL tested once-weekly exenatide and also did not meet conventional superiority criteria, although the point estimate favoured treatment (13). These neutral trials remain important because they prevent simplistic claims of a uniform class effect. The chronological transition from early diabetes-focused cardiovascular safety trials to later expanded cardiometabolic and cardiorenal studies is summarized in **Figure 1A**.

LEADER marked the first decisive demonstration that a GLP-1 RA could reduce cardiovascular events in T2DM. Liraglutide reduced MACE and cardiovascular death in patients with T2DM and high cardiovascular risk (1). SUSTAIN-6, although smaller and shorter, showed a larger relative MACE reduction with subcutaneous semaglutide, driven in part by fewer non-fatal strokes (2). HARMONY Outcomes confirmed cardiovascular benefit with albiglutide in patients with established cardiovascular disease (14), whereas REWIND showed a significant MACE reduction with dulaglutide in a broader population that included a large proportion of patients without established cardiovascular disease (3). AMPLITUDE-O further strengthened the evidence base by demonstrating cardiovascular and kidney benefit with efpeglenatide in high-risk T2DM (15). Representative trial-level estimates for 3-point MACE are shown in **Figure 1B**, illustrating both the consistency of benefit among several long-acting GLP-1 receptor agonists and the neutral findings in selected trials.

**Figure 2:**
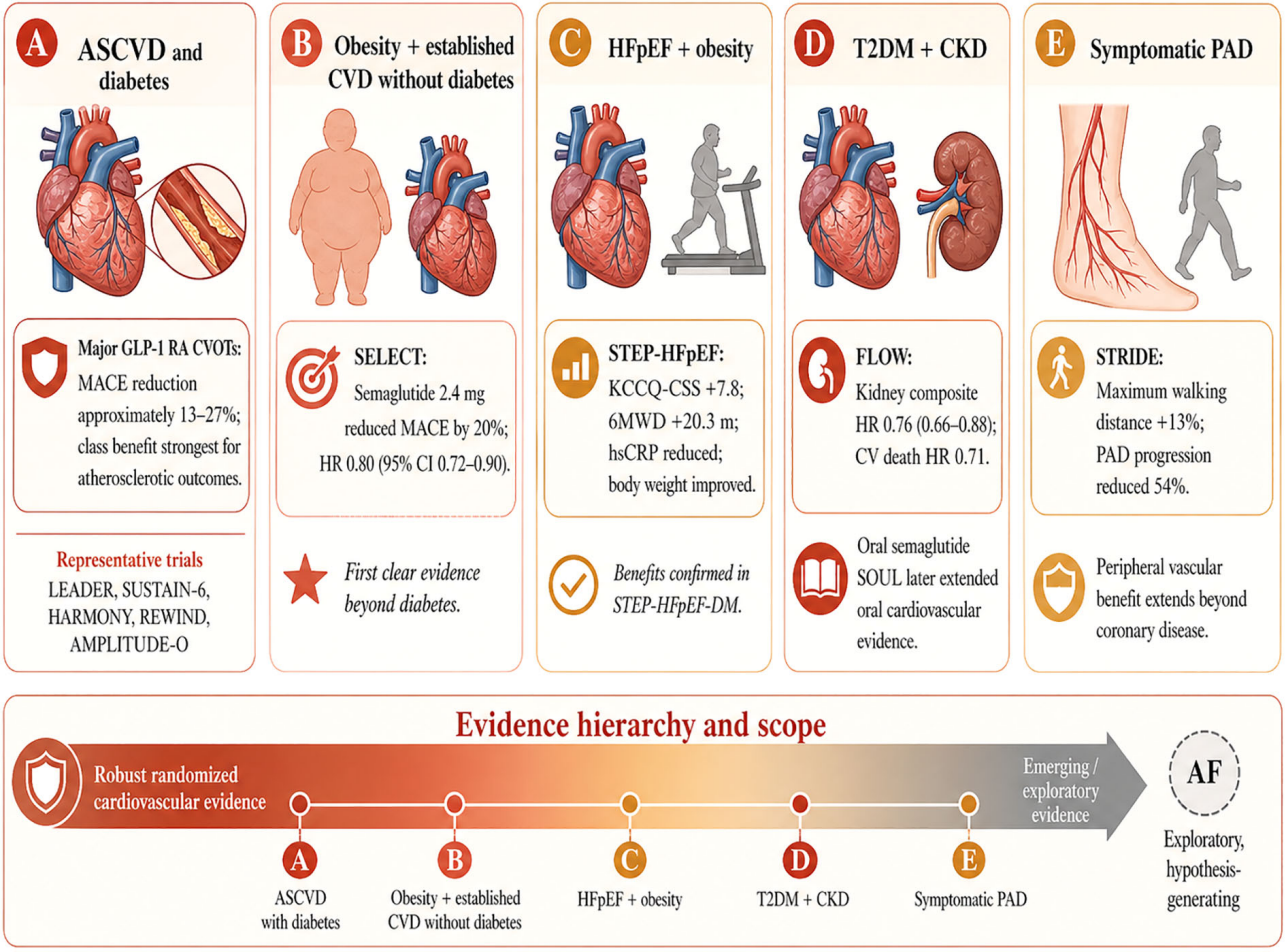
Clinical spectrum and indication-specific benefit of incretin-based therapies. This figure summarizes the major clinical domains in which incretin-based therapies have demonstrated or are developing evidence of cardiovascular, cardiorenal, or functional benefit. In patients with type 2 diabetes and ASCVD or high cardiovascular risk, major GLP-1 receptor agonist CVOTs support reductions in atherosclerotic events, particularly MACE (2A). In patients with overweight or obesity and established cardiovascular disease but without diabetes, SELECT demonstrated that semaglutide 2.4 mg reduced MACE, extending cardiovascular benefit beyond glycemic indications (2B). In obesity-related HFpEF, STEP-HFpEF showed improvements in symptoms, functional status, body weight, and inflammatory biomarkers, with similar benefit later demonstrated in STEP-HFpEF-DM (2C). In T2DM with CKD, FLOW demonstrated kidney protection with cardiovascular and mortality co-benefits, while SOUL extended evidence for oral semaglutide in high-risk patients (2D). In symptomatic PAD, STRIDE demonstrated improved walking capacity and reduced PAD progression (2E). The evidence hierarchy differs across indications: ASCVD and CKD outcomes are supported by robust randomized outcome trials, HFpEF and PAD are supported by diseasespecific functional or cardiorenal trials, whereas atrial fibrillation remains exploratory and hypothesis-generating.

Several themes emerge across these trials. Benefit is generally more evident for atherosclerotic outcomes than for heart-failure hospitalization, the time course is compatible with progressive vascular risk modification rather than an acute anti-ischaemic effect, and the direction of effect is broadly consistent among long-acting agents with adequate systemic exposure (4, 5). However, the magnitude of effect should not be compared naively across trials because populations differed in prior cardiovascular disease prevalence, kidney disease burden, background statin and SGLT2 inhibitor use, follow-up duration, discontinuation rates and endpoint definitions (4, 5, 23). In patients with T2DM and ASCVD or high cardiovascular risk, the most mature evidence remains derived from major GLP-1 RA CVOTs, as summarized in **Figure 2A**.

### 3.2 Oral semaglutide: from PIONEER 6 to SOUL

Oral semaglutide illustrates the distinction between cardiovascular safety and cardiovascular efficacy. PIONEER 6 was designed primarily for safety and had limited power for superiority, producing a favourable but imprecise MACE estimate (28). SOUL subsequently addressed the cardiovascular efficacy question in a much larger high-risk T2DM population and demonstrated superiority of oral semaglutide for MACE reduction (29). This result is clinically important because it establishes that oral GLP-1 RA therapy can deliver outcome benefit, although practical considerations such as fasting requirements, gastrointestinal tolerability, adherence and cost remain central to implementation.

The clinical implication is not that route of administration is irrelevant, but that the route must be judged by exposure, adherence and outcome evidence. Injectable semaglutide, oral semaglutide and emerging small-molecule oral GLP-1 RAs have different pharmacokinetic and behavioural profiles (25, 28, 29). Outcome-proven use should therefore be molecule- and dose-specific rather than extrapolated solely from receptor class. The completed CVOT evidence and key interpretive caveats are summarized in **Table 1**.

**Table 1:** Cardiovascular outcome evidence across major incretin-based trials.

Trial	Agent	Population	Interpretation for cardiovascular practice
ELIXA	Lixisenatide	T2DM after recent ACS; n=6,068	MACE HR 1.02 (0.89-1.17); neutral; established safety after ACS (12)
LEADER	Liraglutide 1.8 mg	T2DM at high CV risk; n=9,340	MACE HR 0.87 (0.78-0.97); CV death reduction; first clear superiority signal (1)
SUSTAIN-6	Semaglutide SC 0.5/1.0 mg	T2DM with CVD or risk factors; n=3,297	MACE HR 0.74 (0.58-0.95); notable stroke signal; retinopathy caveat with rapid HbA1c fall (2)
EXSCEL	Exenatide ER	T2DM with or without established CVD; n=14,752	MACE HR 0.91 (0.83-1.00); did not meet superiority; discontinuation/exposure important (13)
HARMONY Outcomes	Albiglutide	T2DM with established CVD; n=9,463	MACE HR 0.78 (0.68-0.90); strong support for long-acting GLP-1 RA efficacy (14)
REWIND	Dulaglutide 1.5 mg	Broad T2DM population; n=9,901	MACE HR 0.88 (0.79-0.99); most informative trial for broader risk spectrum (3)
PIONEER 6	Oral semaglutide 14 mg	High-risk T2DM; n=3,183	MACE HR 0.79 (0.57-1.11); safety trial, underpowered for superiority (28)
AMPLITUDE-O	Efpeglenatide	T2DM with CVD or CKD; n=4,076	MACE HR 0.73 (0.58-0.92); supports cardiovascular and kidney benefit (15)
SELECT	Semaglutide 2.4 mg	Overweight/obesity plus established CVD without diabetes; n=17,604	MACE HR 0.80 (0.72-0.90); extends benefit beyond diabetes (6, 30)
FLOW	Semaglutide 1.0 mg	T2DM plus CKD; n=3,533	Kidney composite HR 0.76 (0.66-0.88); cardiovascular and mortality co-benefits (7)
SOUL	Oral semaglutide 14 mg	T2DM with ASCVD and/or CKD; n~9,650	MACE HR 0.86 (0.77-0.96); first large oral semaglutide superiority CVOT (29)
SURPASS-CVOT	Tirzepatide vs dulaglutide	T2DM plus ASCVD; n~13,300	Noninferior to dulaglutide for MACE; not proven superior for the primary endpoint (24)

## 4 Beyond diabetes: the non-glycaemic cardiovascular paradigm

The expansion of incretin-based evidence from ASCVD in T2DM to obesity-associated cardiovascular disease, CKD, HFpEF, and PAD is summarized by clinical domain in **Figure 1C**.

### 4.1 SELECT and cardiovascular risk reduction in obesity without diabetes

SELECT was pivotal in extending incretin-based cardiovascular outcome evidence beyond diabetes. In adults with BMI at least 27 kg/m2, established cardiovascular disease and no diabetes, semaglutide 2.4 mg reduced MACE compared with placebo (6). The trial has several strengths: large sample size, adjudicated outcomes, event-driven design, long follow-up and a population in whom glucose lowering was not the therapeutic target. It also has important boundaries. Participants had established cardiovascular disease, so the results do not prove primary-prevention benefit in all patients with obesity. Prespecified analyses suggest consistency across obesity classes, but they do not eliminate the need to individualize treatment according to absolute cardiovascular risk and tolerability (27). The SELECT population and major cardiovascular result are summarized in **Figure 2B**.

The FDA indication for semaglutide 2.4 mg to reduce the risk of cardiovascular death, non-fatal myocardial infarction or non-fatal stroke in adults with established cardiovascular disease and overweight or obesity represents a major regulatory milestone (30). It recognizes obesity as a modifiable cardiovascular risk state rather than merely a lifestyle-associated comorbidity. However, clinical implementation should still be individualized, incorporating absolute risk, tolerability, contraindications, long-term adherence and affordability.

Mechanistically, SELECT strengthens the argument that GLP-1 RA cardiovascular benefit is not simply a consequence of improved glycaemia (6). Reductions in body weight, waist circumference, blood pressure, inflammatory biomarkers and other risk factors likely contributed to benefit, but the absence of diabetes removes HbA1c lowering as a necessary explanation (6, 21). This does not prove a single direct myocardial or vascular mechanism; rather, it supports a multidimensional cardiometabolic model in which adiposity, inflammation, vascular biology and behavioural adherence interact. Subgroup and mediation analyses should therefore be interpreted as mechanistic clues, not as exact causal decomposition (27).

### 4.2 Chronic kidney disease and cardiorenal protection

FLOW was the first dedicated kidney outcomes trial of a GLP-1 RA and demonstrated that semaglutide reduced a composite kidney endpoint in patients with T2DM and chronic kidney disease (7). The trial also reported favourable cardiovascular and mortality outcomes, reinforcing the concept of cardiorenal protection (7). Kidney benefit may be mediated by reductions in albuminuria, systemic inflammation, body weight, blood pressure, oxidative stress and intrarenal inflammatory signalling, but only some of these pathways have been directly tested in humans (31–34). The cardiorenal evidence from FLOW, together with SOUL, is summarized in **Figure 2D**.

The practical consequence is that GLP-1 RAs should be considered part of a layered cardiorenal strategy in high-risk T2DM with CKD, especially when atherosclerotic cardiovascular disease, obesity or persistent albuminuria are present (7, 35). Nevertheless, GLP-1 RA therapy should not displace SGLT2 inhibitors in patients with clear SGLT2 inhibitor indications; available evidence and guideline logic support combination rather than substitution when tolerated (35–37).

### 4.3 Obesity-related HFpEF: symptoms, function and remodelling

Obesity-related HFpEF is a biologically coherent target for incretin therapy because excess adiposity contributes to plasma volume expansion, systemic inflammation, pulmonary vascular load, epicardial fat accumulation, skeletal muscle dysfunction and impaired exercise reserve (38–40). STEP-HFpEF demonstrated that semaglutide 2.4 mg improved symptoms, physical limitations, exercise function and weight in patients with HFpEF and obesity (8). STEP-HFpEF DM extended these findings to patients with obesity-related HFpEF and T2DM (41). These obesity-related HFpEF findings are summarized in **Figure 2C**.

These studies are best interpreted as high-quality evidence for patient-centred functional improvement rather than definitive proof of reduced hard heart-failure events (8, 41). The trials were not primarily powered for cardiovascular death or heart-failure hospitalization. The observed improvements in KCCQ clinical summary score, 6-minute walk distance, body weight and inflammatory biomarkers are clinically meaningful, and imaging substudies suggest favourable cardiac structural changes, but future trials should test event reduction, durability and interaction with SGLT2 inhibitors.

### 4.4 Peripheral artery disease and functional vascular outcomes

STRIDE extended the clinical relevance of semaglutide to symptomatic PAD with T2DM. Semaglutide improved maximum walking distance and reduced disease progression over 52 weeks (9). These findings are important because walking impairment is a patient-centred endpoint directly linked to independence, mobility and quality of life. The PAD signal is consistent with systemic vascular and metabolic effects, but it should not be interpreted as proof that GLP-1 RAs replace antithrombotic therapy, lipid lowering, smoking cessation, exercise therapy or revascularization when indicated. The STRIDE findings in symptomatic PAD are summarized in **Figure 2E**.

The PAD evidence also broadens the meaning of cardiovascular benefit beyond MACE (9). Event prevention, kidney protection, symptom improvement, exercise capacity, and quality of life should be distinguished because these domains carry different implications for patients, clinicians, regulators, and payers.

## 5 Molecular and physiological mechanisms

### 5.1 Receptor signalling and tissue-level biology

**Figure 3:**
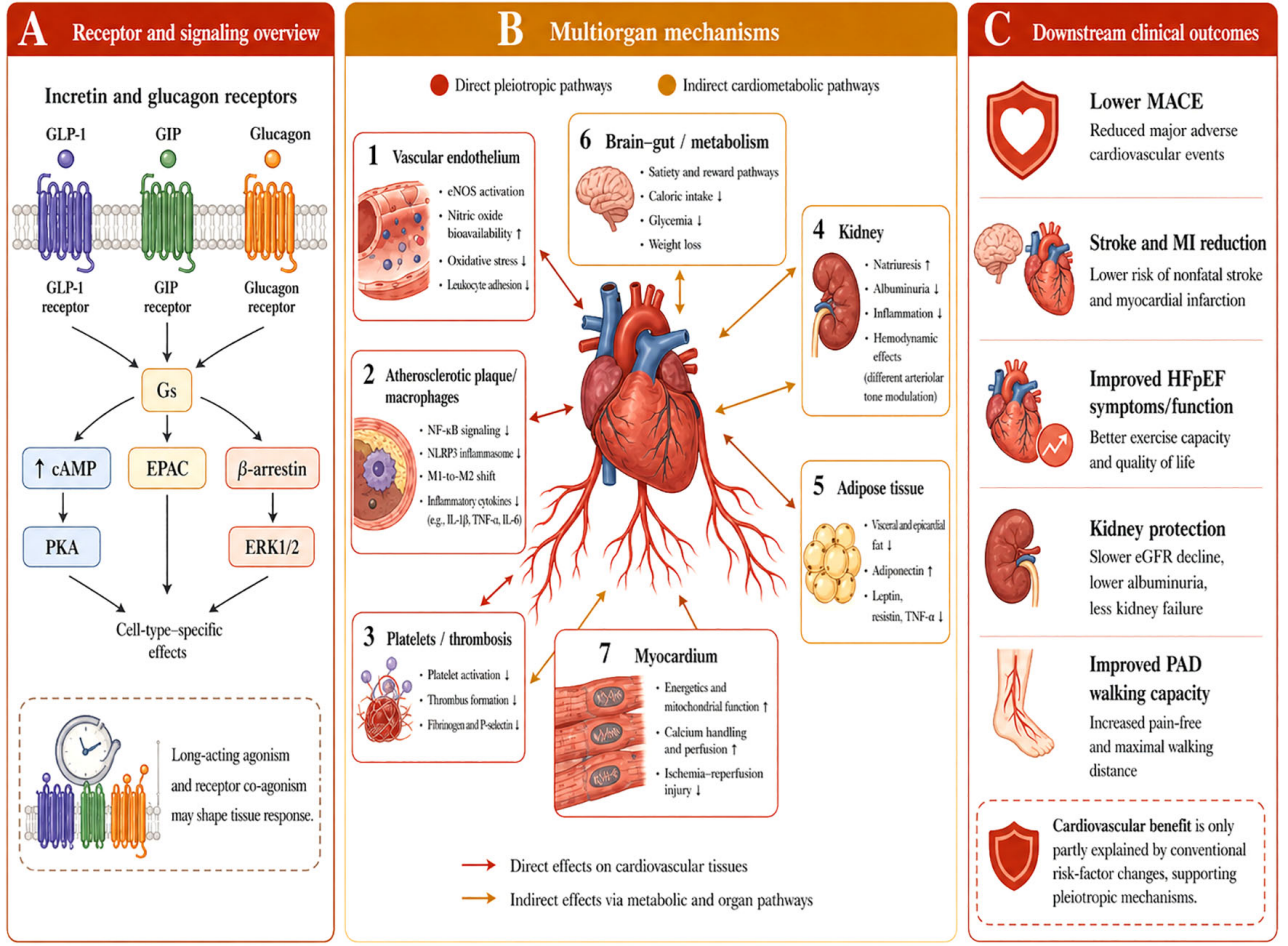
Multiorgan mechanisms of incretin-mediated cardiovascular protection. This image integrates receptor-level signalling with tissue-specific and systemic pathways through which incretin-based therapies may confer cardiovascular and cardiorenal benefit. **(A)** Summarizes GLP-1, GIP, and glucagon receptor signalling through Gs-cAMP-PKA, EPAC, and β-arrestin/ERK pathways, with potential modulation by long-acting receptor engagement and receptor co-agonism. **(B)** Depicts multiorgan mechanisms involving the vascular endothelium, atherosclerotic plaque and macrophage biology, platelets and thrombosis, kidney, adipose tissue, brain–gut–metabolic pathways, and myocardium. These mechanisms include improved endothelial nitric oxide bioavailability, reduced oxidative stress, attenuation of NF-κB/NLRP3-mediated inflammation, altered macrophage polarization, reduced platelet activation, natriuresis, albuminuria reduction, reduction of visceral and epicardial adiposity, appetite and weight regulation, and potential myocardial protection. **(C)** Links these pathways to clinically observed outcomes, including lower MACE, reduced stroke and myocardial infarction, improved HFpEF symptoms and functional capacity, kidney protection, and improved PAD walking capacity. The relative contribution of each pathway in humans remains incompletely quantified and likely varies by agent, dose, exposure duration, tissue context, and patient phenotype. cAMP, cyclic adenosine monophosphate; eGFR, estimated glomerular filtration rate; eNOS, endothelial nitric oxide synthase; EPAC, exchange protein directly activated by cAMP; ERK, extracellular signal-regulated kinase; GIP, glucose-dependent insulinotropic polypeptide; GLP-1, glucagon-like peptide-1; HFpEF, heart failure with preserved ejection fraction; IL, interleukin; MACE, major adverse cardiovascular events; MI, myocardial infarction; NF-κB, nuclear factor kappa B; NLRP3, nucleotide-binding oligomerization domain-like receptor family pyrin domain-containing 3 inflammasome; PAD, peripheral arterial disease; PKA, protein kinase A; TNF-α, tumour necrosis factor-α.

The GLP-1 receptor is a class B G-protein-coupled receptor that signals predominantly through Gs-mediated activation of adenylyl cyclase, cyclic AMP, protein kinase A and EPAC (16–18). In pancreatic beta cells, this pathway enhances glucose-dependent insulin secretion and beta-cell survival (16–18). In vascular and immune contexts, cyclic AMP-dependent signalling can suppress inflammatory transcriptional programmes, improve endothelial nitric oxide bioavailability, reduce oxidative stress and alter macrophage phenotype (19, 20). The degree to which these mechanisms operate in humans at clinically used doses remains an active area of translational investigation. The receptor-level signalling architecture, including GLP-1, GIP, and glucagon receptor pathways, is summarized in **Figure 3A**.

Cardiovascular protection is unlikely to reflect a single dominant pathway. Instead, incretin therapy generates a network effect across adipose tissue, vasculature, kidney, liver, autonomic regulation, inflammation, platelet function, skeletal muscle and central satiety pathways (16–19, 31). Mechanistic interpretation requires distinction among proximal pharmacology, intermediate phenotypes, and clinical outcomes. Receptor activation is proximal; weight loss, blood-pressure reduction, hsCRP decline, albuminuria reduction and lower triglycerides are intermediate; fewer cardiovascular events are the outcome. Causality cannot be inferred simply because an intermediate changes, but convergent evidence across mechanistic and clinical layers strengthens plausibility. The major mechanistic pathways are summarized in **Table 2** and **Figure 3B**.

**Table 2:** Mechanistic pathways and evidentiary interpretation.

Pathway	Biological signal	Evidence interpretation
Inflammation	Lower hsCRP; reduced NF-kappaB/NLRP3 signalling; altered macrophage phenotype	Supported by biomarker and experimental data; independent clinical contribution not precisely quantified (19, 21)
Endothelium	Improved nitric oxide bioavailability; lower oxidative stress; reduced leukocyte adhesion	Mechanistically plausible; strongest causal data are experimental rather than human outcome-specific (19, 20)
Atherothrombosis	Reduced platelet activation and thrombosis in experimental systems	Consistent with MACE reductions; no dedicated platelet-driven clinical endpoint trial (43)
Adipose tissue	Reduced visceral and epicardial fat; altered adipokines and inflammatory secretome	Relevant to ASCVD, HFpEF and AF; difficult to separate from total weight loss (22, 38, 39)
Kidney	Albuminuria reduction; natriuresis; anti-inflammatory and haemodynamic effects	FLOW provides dedicated kidney outcome evidence for semaglutide in T2DM with CKD (7)
Central and autonomic pathways	Satiety, reduced caloric intake, possible autonomic effects	Important for weight loss; cardiovascular causal contribution remains under study
Skeletal muscle/function	Weight loss may improve mobility but can include lean mass loss	Requires resistance exercise and nutritional strategies in older or frail patients

### 5.2 Inflammation and atherosclerotic plaque biology

Inflammation is a leading candidate pathway for cardiovascular benefit beyond classical risk-factor modification. Semaglutide and other GLP-1 RAs reduce high-sensitivity C-reactive protein, with some reduction occurring early during treatment before maximal weight loss (21). Experimental evidence suggests inhibition of NF-kappaB activity, NLRP3 inflammasome activation and pro-inflammatory cytokine release (19). GLP-1 receptor activation may also promote a shift from pro-inflammatory to reparative macrophage phenotypes and reduce monocyte-endothelial adhesion (19). These effects are consistent with the delayed separation of MACE curves in several CVOTs, which is compatible with progressive stabilization of atherosclerotic lesions rather than acute vasodilation alone (5, 42).

The evidence should nevertheless be framed cautiously. Human plaque-level data are limited, and circulating hsCRP is an imperfect surrogate for tissue inflammation. Moreover, weight loss itself reduces inflammatory burden (21). The most defensible conclusion is that anti-inflammatory effects likely contribute to cardiovascular benefit, but their independent quantitative contribution remains uncertain.

### 5.3 Endothelial, platelet and haemodynamic pathways

Endothelial dysfunction is a central feature of atherosclerosis, diabetes, obesity and CKD. Experimental studies support GLP-1 receptor-dependent improvement in endothelial nitric oxide signalling, reduction in oxidative stress and attenuation of vascular inflammation (19, 20). Platelet studies suggest that GLP-1 receptor activation can reduce platelet aggregation through nitric oxide/cyclic GMP-dependent mechanisms, providing a plausible anti-thrombotic component (43). These mechanisms align with reductions in myocardial infarction and stroke observed across meta-analyses, although dedicated thrombosis endpoint trials are lacking (5).

Haemodynamic effects are modest but clinically relevant at population scale. GLP-1 RAs lower systolic blood pressure by approximately 2–5 mmHg in many studies, promote natriuresis through renal tubular pathways, and reduce arterial stiffness in some mechanistic analyses (31, 44). They also increase resting heart rate by a small amount, typically 2–4 beats per minute (45). Whether this chronotropic effect has clinical significance in arrhythmia-prone populations remains uncertain, but it justifies monitoring in patients with atrial fibrillation, advanced heart failure or ventricular arrhythmia substrates.

### 5.4 Adipose tissue, epicardial fat and cardiac mechanics

Visceral and epicardial adipose tissues are not passive energy stores. They secrete inflammatory cytokines, adipokines and profibrotic mediators and are anatomically linked to coronary arteries and atrial myocardium (22, 38, 39). GLP-1 RA-induced weight loss preferentially reduces visceral adiposity in many studies, and reductions in epicardial fat may plausibly improve coronary microvascular function, atrial substrate and diastolic mechanics (22, 46). This biology is particularly relevant to obesity-related HFpEF and atrial fibrillation (38, 46, 47).

Skeletal muscle also matters. High-efficacy weight loss can improve mobility, insulin sensitivity and inflammatory burden, but excessive loss of lean mass may reduce functional reserve in older adults, patients with HFpEF, sarcopenic obesity or PAD (48, 49). Cardiovascular prescribing of incretin therapy should therefore be coupled with resistance exercise, adequate protein intake and functional monitoring, particularly when using high-potency agents or when weight loss exceeds 15-20%. The downstream clinical correlates of these multiorgan pathways are summarized in **Figure 3C**.

## 6 Multi-agonist and oral small-molecule therapeutics

### 6.1 Tirzepatide and the meaning of active-comparator evidence

Tirzepatide is a dual glucose-dependent insulinotropic polypeptide (GIP) and GLP-1 receptor agonist that produces greater weight loss and glycaemic improvement than several GLP-1 RA comparators in metabolic trials (50, 51). The cardiovascular question, however, requires adjudicated outcomes. SURPASS-CVOT tested tirzepatide against dulaglutide in patients with T2DM and established atherosclerotic cardiovascular disease (24). This active-comparator design is scientifically demanding because the control treatment itself has cardiovascular outcome benefit (3).

The primary interpretation is that tirzepatide was noninferior to dulaglutide for MACE but did not prove superiority for the primary endpoint (24). This distinction is critical for scientific accuracy. Secondary outcomes, including mortality and kidney signals, may be clinically important, but they must be interpreted in the context of the statistical hierarchy (24). The trial supports cardiovascular safety and comparable efficacy versus an outcome-proven GLP-1 RA; it does not establish tirzepatide as superior to dulaglutide for MACE prevention (24). Dedicated data in obesity without diabetes, HFpEF and CKD populations will be needed to define whether the greater weight-loss efficacy of dual agonism translates into incremental cardiovascular event reduction.

### 6.2 Retatrutide, CagriSema and receptor expansion

Retatrutide is a triple GLP-1/GIP/glucagon receptor agonist that has produced very large weight reductions in phase 2 obesity studies (52). Glucagon receptor agonism may enhance energy expenditure and hepatic lipid metabolism, potentially complementing central satiety and insulinotropic pathways. CagriSema combines semaglutide with the amylin analogue cagrilintide, targeting appetite, satiety and weight through partially distinct neuroendocrine pathways (53). These agents illustrate the next phase of incretin-based pharmacology: increasing metabolic potency through receptor combinations.

Cardiovascular interpretation should remain conservative until outcome trials are completed (52, 53). More weight loss is likely to improve cardiometabolic risk factors, but very large and rapid weight reduction may introduce new clinical issues, including lean mass loss, gallbladder disease, nutritional deficiency, dose discontinuation and uncertain effects in frail or older cardiovascular patients. Future CVOTs should therefore include hard cardiovascular events, kidney outcomes, arrhythmia surveillance, body-composition substudies, patient-reported outcomes and long-term discontinuation analyses.

### 6.3 Orforglipron and oral non-peptide GLP-1 receptor agonism

Orforglipron is an oral, non-peptide small-molecule GLP-1 receptor agonist (25). As of 30 April 2026, FDA documents list Foundayo (orforglipron) as approved for chronic weight management in adults with obesity or overweight with at least one weight-related comorbidity, in combination with reduced caloric intake and increased physical activity (26). This is a major delivery innovation because small-molecule oral therapy may reduce injection barriers, cold-chain constraints and administration complexity.

**Figure 4:**
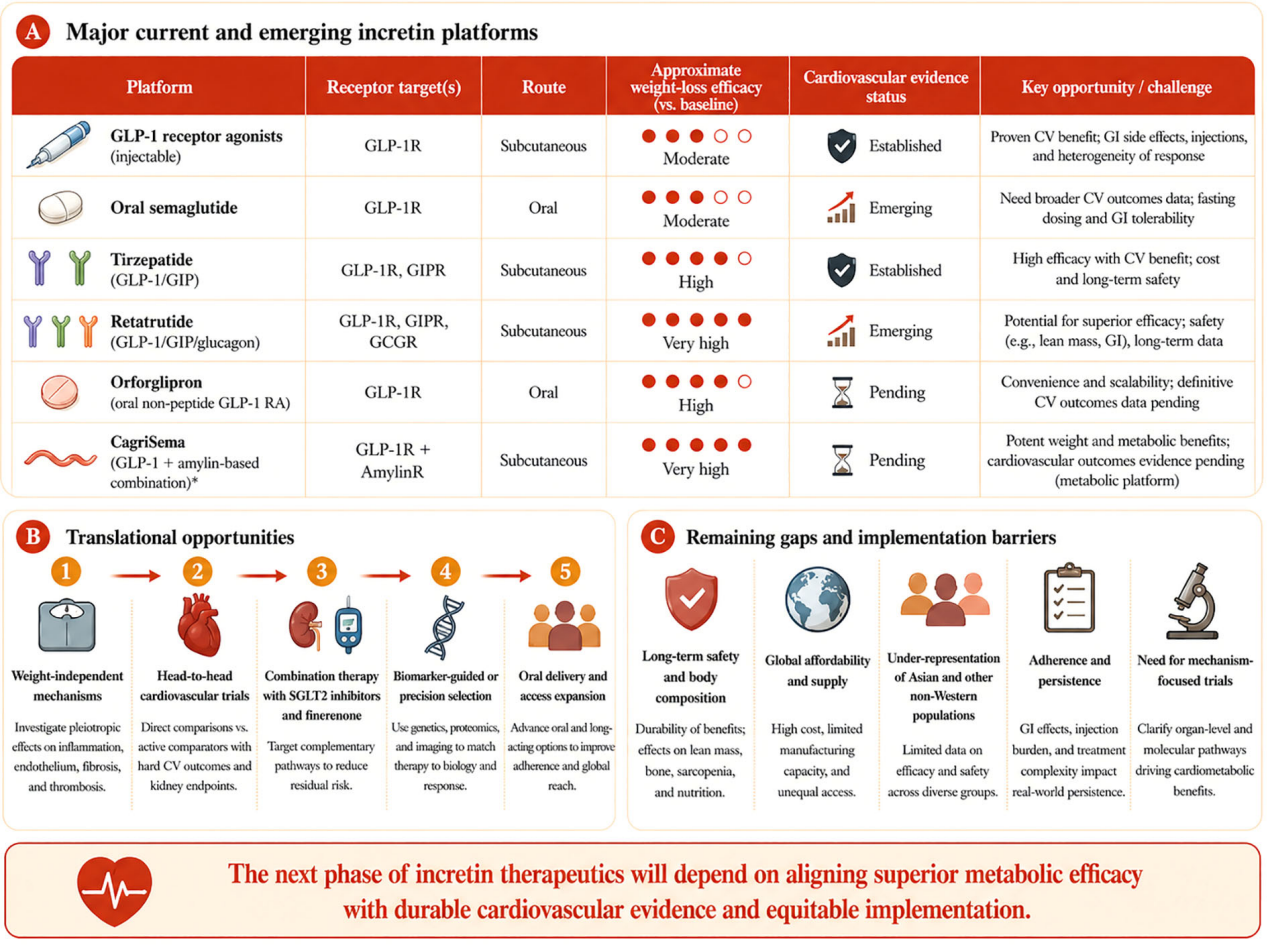
Next-generation incretin therapeutics, translational opportunities, and remaining gaps This image summarizes the emerging therapeutic landscape and future research priorities for incretin-based cardiovascular medicine. **(A)** Compares current and emerging incretin platforms by receptor target, route of administration, approximate weight-loss efficacy, cardiovascular evidence status, and key opportunities or challenges. Established injectable GLP-1 receptor agonists have the strongest cardiovascular outcomes evidence, whereas oral semaglutide expands delivery options but requires continued evaluation across broader populations. Tirzepatide provides strong metabolic efficacy with evolving comparative cardiovascular positioning, while retatrutide, orforglipron, and amylin-based combination strategies may broaden efficacy, convenience, or scalability but require definitive cardiovascular outcomes and long-term safety data. **(B)** Outlines translational priorities, including clarification of weight-independent mechanisms, head-to-head cardiovascular trials, rational combination therapy with SGLT2 inhibitors and finerenone, biomarker-guided patient selection, and oral or long-acting delivery strategies to improve access and persistence. **(C)** Highlights unresolved barriers, including long-term safety and body composition effects, affordability and supply, under-representation of Asian and other non-Western populations, adherence and treatment persistence, and the need for mechanism-focused human studies. The next phase of the field will depend on aligning superior metabolic efficacy with durable cardiovascular evidence, mechanistic clarity, and equitable implementation. CV, cardiovascular; GCGR, glucagon receptor; GI, gastrointestinal; GIP, glucose-dependent insulinotropic polypeptide; GIPR, glucose-dependent insulinotropic polypeptide receptor; GLP-1 RA, glucagon-like peptide-1 receptor agonist; GLP-1R, glucagon-like peptide-1 receptor; SGLT2, sodium-glucose cotransporter-2.

The cardiovascular conclusion must be precise: approval for weight management is not equivalent to proven cardiovascular outcome benefit (26). Orforglipron has shown weight-loss and risk-factor effects, but adjudicated cardiovascular outcome data are needed before it can be positioned alongside semaglutide 2.4 mg for cardiovascular risk reduction in established CVD (25, 26). A major potential contribution of oral non-peptide GLP-1 RAs may be implementation: lower manufacturing complexity, easier distribution and improved patient acceptance could expand access if pricing and coverage are aligned with population health needs. The therapeutic evolution and evidence status of next-generation agents are summarized in **Table 3** and visually compared in **Figure 4A**.

**Table 3:** Next-generation incretin-based therapeutics: evidence status and clinical caution.

Agent	Target profile	Administration	Cardiovascular interpretation
Semaglutide 2.4 mg	GLP-1 RA	Weekly injection	SELECT outcome benefit in obesity plus established CVD without diabetes; FDA CV risk-reduction indication (6, 30)
Oral semaglutide 14 mg	GLP-1 RA	Daily oral peptide with absorption enhancer	SOUL demonstrates MACE reduction in high-risk T2DM; administration constraints affect adherence (29)
Tirzepatide	Dual GIP/GLP-1 agonist	Weekly injection	SURPASS-CVOT: noninferior to dulaglutide for MACE; superiority not established for primary endpoint (24)
Retatrutide	GLP-1/GIP/glucagon agonist	Weekly injection	Very high weight-loss efficacy in phase 2; CVOT evidence pending (52)
CagriSema	GLP-1/amylin combination	Weekly injection	High weight-loss efficacy; cardiovascular outcomes require dedicated study (53)
Orforglipron/Foundayo	Oral non-peptide GLP-1 RA	Daily oral small molecule	FDA-approved in 2026 for chronic weight management; no completed CVOT-based CV indication (25, 26)

## 7 Clinical decision framework

**Figure 5:**
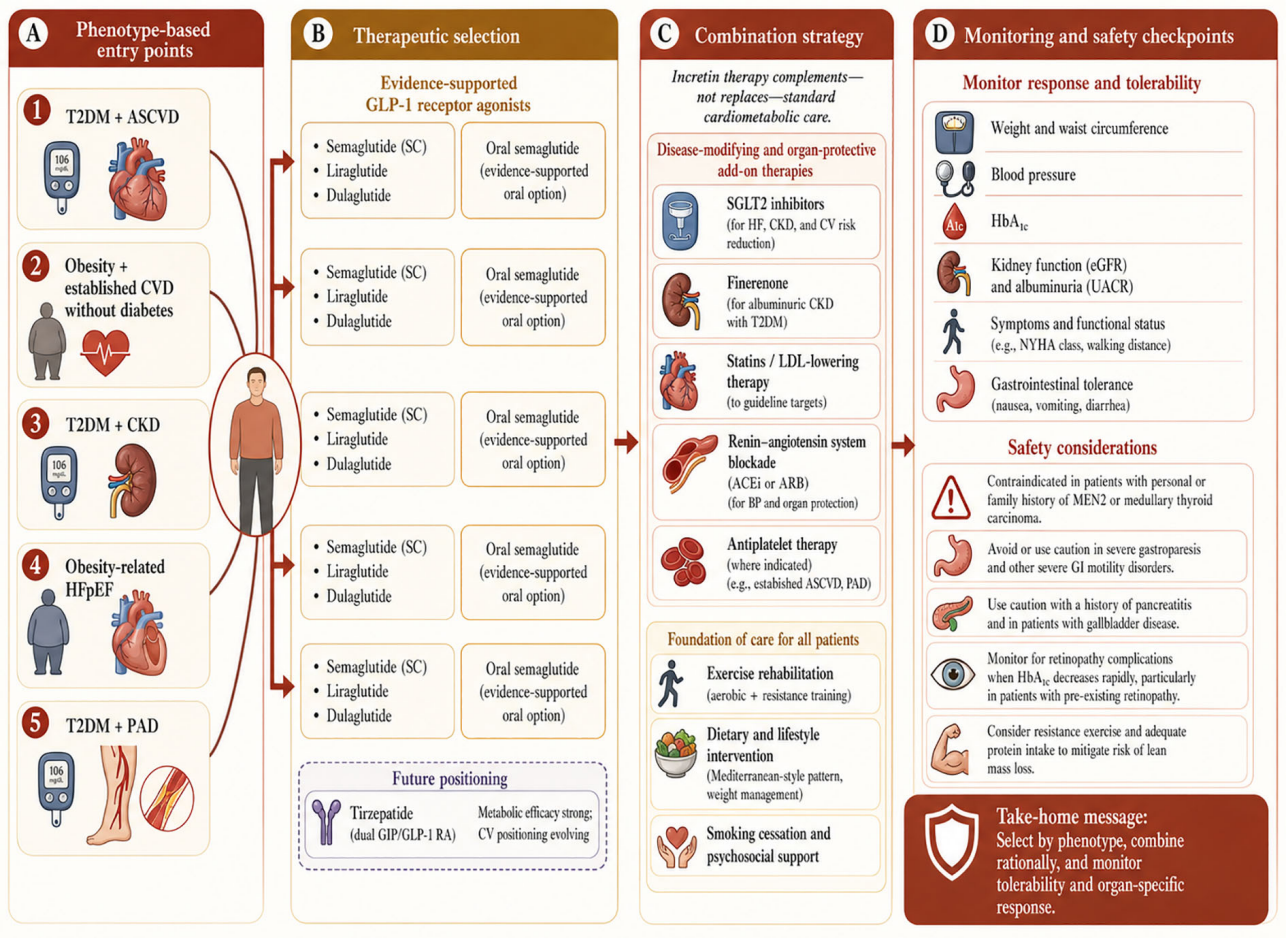
Clinical decision pathway for incretin therapy in cardiometabolic disease. This image proposes a phenotype-based clinical decision framework for integrating incretin-based therapy into cardiometabolic care. **(A)** Identifies common entry phenotypes, including T2DM with ASCVD, obesity with established cardiovascular disease without diabetes, T2DM with CKD, obesity-related HFpEF, and T2DM with PAD. **(B)** Summarizes evidence-supported incretin options, including established GLP-1 receptor agonists and oral semaglutide where supported by outcome data, while positioning tirzepatide as a metabolically potent agent whose comparative cardiovascular role continues to evolve. **(C)** Emphasizes that incretin therapy should complement, rather than replace, guideline-directed cardiometabolic and organ-protective care, including SGLT2 inhibitors, finerenone in albuminuric CKD, LDL-lowering therapy, renin–angiotensin system blockade, antiplatelet therapy when indicated, exercise rehabilitation, dietary intervention, smoking cessation, and psychosocial support. **(D)** Summarizes response monitoring and safety checkpoints, including body weight, waist circumference, blood pressure, HbA1c, kidney function, albuminuria, functional status, gastrointestinal tolerability, contraindications related to medullary thyroid carcinoma or MEN2, caution in severe gastroparesis, pancreatitis or gallbladder disease, retinopathy risk during rapid glycaemic improvement, and lean mass preservation strategies. The framework is intended as a conceptual clinical synthesis rather than a substitute for local regulatory labelling or guideline-directed individualized decision-making. ACEi, angiotensin-converting enzyme inhibitor; ARB, angiotensin receptor blocker; ASCVD, atherosclerotic cardiovascular disease; BP, blood pressure; CKD, chronic kidney disease; CV, cardiovascular; CVD, cardiovascular disease; eGFR, estimated glomerular filtration rate; GI, gastrointestinal; GIP, glucose-dependent insulinotropic polypeptide; GLP-1 RA, glucagon-like peptide-1 receptor agonist; HbA1c, glycated haemoglobin; HF, heart failure; HFpEF, heart failure with preserved ejection fraction; LDL, low-density lipoprotein; MEN2, multiple endocrine neoplasia type 2; MTC, medullary thyroid carcinoma; NYHA, New York Heart Association; PAD, peripheral arterial disease; SC, subcutaneous; SGLT2, sodium-glucose cotransporter-2; T2DM, type 2 diabetes mellitus; UACR, urine albumin-to-creatinine ratio.

A phenotype-based entry framework for incretin therapy is shown in **Figure 5A**, including T2DM with ASCVD, established CVD with overweight or obesity without diabetes, T2DM with CKD, obesity-related HFpEF, and T2DM with PAD.

### 7.1 ASCVD with type 2 diabetes

For patients with T2DM and established ASCVD, GLP-1 RAs with proven cardiovascular benefit - particularly liraglutide, subcutaneous semaglutide and dulaglutide, with additional evidence for efpeglenatide where available - should be considered independent of baseline HbA1c when cardiovascular risk reduction is a therapeutic goal (1–3, 54, 55). This approach aligns with contemporary diabetes and cardiovascular guidelines that prioritize comorbidity-directed therapy rather than glucose-centric escalation alone (54–56).

Selection among agents should consider outcome evidence, weight goals, kidney disease, route preference, gastrointestinal tolerability, retinopathy status, cost and formulary access (54–56). When heart failure or CKD predominates, SGLT2 inhibitors remain foundational; when ASCVD, obesity or stroke risk predominates, an outcome-proven GLP-1 RA is particularly attractive (35, 36, 54). In many high-risk patients, the most evidence-consistent approach is combination therapy rather than sequential substitution (36, 37). Evidence-supported therapeutic options and areas where comparative cardiovascular positioning remains evolving are summarized in **Figure 5B**.

### 7.2 Established CVD with overweight or obesity but without diabetes

In adults with established cardiovascular disease and overweight or obesity without diabetes, semaglutide 2.4 mg is the key outcome-proven incretin therapy (6, 30). The target population should resemble SELECT: established CVD and BMI at least 27 kg/m2 (6). Clinical decisions should include assessment of absolute cardiovascular risk, obesity-related complications, prior pancreatitis or gallbladder disease, gastrointestinal tolerance, patient preference, treatment affordability and ability to sustain long-term therapy.

This indication may substantially influence the organization of cardiovascular prevention clinics. Cardiologists who historically referred obesity pharmacotherapy to endocrinology or weight-management clinics will increasingly need to understand dose escalation, adverse-event management, peri-procedural interruption, nutritional counselling and long-term monitoring (30, 56). Multidisciplinary care is especially important for patients with frailty, sarcopenia, eating disorders, advanced CKD or recurrent dehydration.

### 27.3 HFpEF and obesity

Obesity-related HFpEF should be approached as a systemic cardiometabolic syndrome rather than isolated ventricular stiffness (38–40). Semaglutide 2.4 mg improves symptoms and exercise function in this phenotype and should be considered in appropriate patients alongside SGLT2 inhibitors, diuretics, blood-pressure control, atrial fibrillation management, sleep apnoea treatment and exercise rehabilitation (8, 41). The strongest evidence is for symptomatic and functional improvement; event reduction remains under investigation (8, 41).

A practical strategy is to define the dominant treatment objective at baseline: decongestion, functional limitation, obesity-related inflammation, glycaemic control, atrial fibrillation, renal congestion or frailty. Dose escalation should be slower in patients with borderline volume status or high diuretic requirements because nausea, reduced intake and vomiting can precipitate dehydration, renal function decline or orthostatic symptoms.

### 7.4 CKD, albuminuria and layered cardiorenal therapy

In T2DM with CKD, semaglutide has dedicated kidney outcome evidence from FLOW (7). Treatment should be layered with renin-angiotensin system blockade, SGLT2 inhibitors and finerenone when indicated and tolerated (35, 37). Albuminuria, eGFR trajectory, hyperkalaemia risk, blood pressure, volume status and patient preference should guide sequencing. The concept of layered therapy is particularly important because residual risk remains high even when one pathway is addressed.

Combination therapy raises practical questions: how to sequence agents, how to manage early eGFR changes, how to avoid hypotension or dehydration, and how to support adherence to multiple long-term drugs (35, 37). In stable patients, SGLT2 inhibitor initiation may be prioritized for rapid kidney and heart-failure protection, followed by GLP-1 RA initiation for ASCVD, weight and albuminuria benefit (35–37). In patients with severe obesity or prior stroke, GLP-1 RA initiation may be prioritized earlier.

### 7.5 PAD and vascular functional limitation

In PAD with T2DM, semaglutide has evidence for improving walking capacity (9). It should be viewed as an adjunct to comprehensive vascular care, not a substitute. The essential background includes high-intensity statin therapy, antithrombotic therapy when indicated, smoking cessation, blood-pressure control, structured exercise therapy, foot care and revascularization evaluation for lifestyle-limiting claudication or chronic limb-threatening ischaemia (57). The indication-based clinical framework is summarized in **Table 4**.

**Table 4:** Practical indication-based use of incretin therapy in cardiovascular medicine.

Clinical phenotype	Preferred treatment logic	Implementation caveat
T2DM + established ASCVD	Outcome-proven GLP-1 RA; combine with SGLT2 inhibitor when CKD/HF risk is present	Avoid HbA1c-only decision-making; consider retinopathy if rapid HbA1c fall expected
Established CVD + overweight/obesity without diabetes	Semaglutide 2.4 mg when SELECT-like criteria are met	Evidence is secondary prevention; do not extrapolate to all obesity patients (6, 30)
Obesity-related HFpEF	Semaglutide 2.4 mg for symptoms/function; optimize SGLT2 inhibitor and diuretics	Hard HF event reduction not yet definitive; monitor volume status
T2DM + CKD/albuminuria	Layer GLP-1 RA with SGLT2 inhibitor, RAS blockade and finerenone when indicated	Monitor eGFR, UACR, dehydration, hyperkalaemia and tolerability
T2DM + symptomatic PAD	Semaglutide as adjunct to vascular prevention and exercise therapy	Functional endpoints improved; limb-event evidence remains incomplete
High frailty/sarcopenia risk	Individualize dose escalation; prescribe resistance exercise and protein optimization	Co-prescribe resistance exercise and adequate protein; monitor frail older adults and sarcopenic obesity (48, 49)

The functional nature of PAD endpoints also makes adherence support essential (9). Patients may experience gastrointestinal symptoms during dose escalation and require encouragement to continue supervised or home-based walking programmes. Weight loss may reduce joint load and improve mobility, creating synergy with exercise rehabilitation.

## 8 Safety, tolerability and monitoring

The safety profile of GLP-1 RAs is well characterized, but cardiovascular clinicians must manage adverse effects proactively to improve persistence (45). Gastrointestinal symptoms are the most common reason for dose interruption or discontinuation (45). Nausea, vomiting, diarrhoea, constipation and abdominal discomfort are usually dose-related and most frequent during escalation. Slower titration, smaller meals, avoidance of high-fat meals during escalation, hydration counselling and temporary dose holds can substantially improve tolerability. Practical monitoring and safety checkpoints for cardiometabolic prescribing are summarized in **Figure 5D**.

Gallbladder and biliary disease risk is increased, particularly with larger and faster weight loss (58). Patients should be counselled regarding right upper quadrant pain, fever, jaundice and persistent vomiting. Pancreatitis has not shown a consistent major excess signal in randomized trial meta-analyses, but a history of pancreatitis remains a reason for cautious individualized prescribing (4, 45). GLP-1 RAs are generally avoided in patients with a personal or family history of medullary thyroid carcinoma or multiple endocrine neoplasia type 2 because of the class boxed warning based largely on rodent C-cell findings and regulatory precaution (45, 59).

Diabetic retinopathy deserves special attention in patients with pre-existing retinopathy and rapid HbA1c improvement, particularly with semaglutide (2). The SUSTAIN-6 retinopathy signal is most plausibly related to rapid glycaemic improvement in susceptible individuals rather than a direct retinal toxicity, but ophthalmological assessment and gradual glycaemic improvement remain prudent (2). In non-diabetic SELECT-like patients, this concern is less relevant (6).

Peri-procedural management has become increasingly important because delayed gastric emptying may increase aspiration risk during sedation or anaesthesia (45). Recommendations continue to evolve, but clinicians should document GLP-1 RA use, dose escalation status, gastrointestinal symptoms and timing of the most recent dose before elective procedures. Coordination with anaesthesia teams is essential, particularly in patients with active nausea, vomiting, gastroparesis, high-dose therapy or recent escalation.

Long-term treatment raises additional issues beyond classical adverse events. Weight regain after discontinuation is common, indicating that obesity pharmacotherapy often requires chronic therapy (49). Access interruptions caused by shortages or cost may therefore produce recurrent weight cycling and risk-factor instability. In high-efficacy regimens, body-composition monitoring is increasingly important (48, 49). Resistance exercise, adequate dietary protein and physical-function assessment should be incorporated into cardiovascular prevention rather than treated as optional lifestyle advice. Key safety domains and monitoring strategies are summarized in **Table 5**.

**Table 5:** Safety domains relevant to cardiovascular prescribing.

Domain	Clinical signal	Management approach
Gastrointestinal intolerance	Nausea, vomiting, diarrhoea, constipation; dose-related	Slow titration; meal-size counselling; hydration; temporary dose hold; avoid in severe gastroparesis (45)
Gallbladder/biliary disease	Cholelithiasis and cholecystitis, partly weight-loss related	Counsel symptoms; evaluate persistent abdominal pain; individualize in prior biliary disease (58)
Pancreatitis	No consistent large RCT signal, but residual uncertainty	No consistent major RCT signal; use individualized caution after prior pancreatitis (4, 45)
Thyroid C-cell warning	Rodent signal; human causality not established	Avoid in personal/family history of medullary thyroid carcinoma or MEN2; human observational signal remains uncertain (45, 59)
Diabetic retinopathy	Signal with rapid HbA1c fall in susceptible patients	Baseline eye assessment in established retinopathy; avoid abrupt glycaemic drops when possible
Heart rate increase	Small resting HR increase, usually 2–4 bpm	Monitor in AF, advanced heart failure or arrhythmia-prone patients (45)
Lean mass loss	Part of large weight reduction may be lean tissue	Co-prescribe resistance exercise and adequate protein; monitor frail older adults and sarcopenic obesity (48, 49)
Peri-procedural gastric emptying	Potential aspiration risk with active symptoms or recent escalation	Communicate with anaesthesia; individualized holding strategy based on risk

## 9 Guidelines, regulation and implementation

Guidelines have rapidly moved from glycaemic algorithms toward comorbidity-directed therapy (54–56). The 2023 ESC diabetes and cardiovascular disease guideline and contemporary ADA Standards of Care recommend GLP-1 RAs with proven cardiovascular benefit for patients with T2DM and ASCVD or high cardiovascular risk, independent of HbA1c in appropriate clinical contexts (54, 55). After SELECT, cardiovascular societies increasingly recognize semaglutide 2.4 mg for overweight or obese patients with established CVD even without diabetes, while FDA labelling provides the regulatory basis in the United States (30, 57).

WHO issued its first global guideline on GLP-1-based therapies for adult obesity in December 2025, conditionally recommending long-term use of GLP-1 therapies as part of comprehensive obesity care while emphasizing access, affordability and health-system readiness (60). This global perspective is essential because the evidence base is strongest in high-income settings, whereas obesity and cardiometabolic disease burden are rising rapidly worldwide, including in low- and middle-income countries (61). Without deliberate access strategies, incretin therapy could widen rather than narrow cardiovascular disparities (60, 62).

Implementation barriers are substantial. Real-world persistence with GLP-1 RAs is often poor, especially when treatment is prescribed as a weight-loss intervention rather than a chronic disease-modifying therapy (62, 63). Discontinuation is driven by gastrointestinal adverse effects, cost, shortages, unrealistic expectations, limited follow-up, and stigma surrounding obesity pharmacotherapy. Effective clinical translation requires systematic follow-up during dose escalation, patient education emphasizing cardiovascular and functional benefits, insurance navigation, nutrition and exercise support, and transition plans during drug shortages.

Equity considerations should be built into prescribing frameworks. Black, Hispanic, Asian and lower-income patients may face disproportionate barriers related to insurance coverage, clinician prescribing patterns, language access, supply distribution and specialist referral (62). East Asian populations also have distinct cardiometabolic phenotypes, including higher visceral adiposity at lower BMI and different diabetes pathophysiology (64, 65). Future trials should include adequate Asian representation and prespecified subgroup analyses by ethnicity, BMI category, body composition and kidney disease phenotype.

### 9.1 Meta-analytic integration and heterogeneity

Meta-analyses provide the best summary of average treatment effects, but they should be used to clarify rather than obscure heterogeneity. Across randomized GLP-1 RA trials in T2DM, pooled estimates generally show reductions in MACE, cardiovascular death and all-cause mortality, with more modest and less consistent effects on heart-failure hospitalization (4, 5, 66). The direction of effect is reassuring across several long-acting molecules, yet neutral trials remain part of the evidence base (5, 12, 13). A mature interpretation therefore recognizes a favourable class tendency among agents with sustained systemic exposure, while preserving molecule-specific distinctions.

The key sources of heterogeneity are clinically meaningful. Patients with established ASCVD differ from those with risk factors alone; CKD populations have higher absolute event rates and competing kidney outcomes; older trials had lower background use of SGLT2 inhibitors and contemporary lipid-lowering therapy; and obesity trials use higher semaglutide doses than diabetes CVOTs (4–7). These differences influence absolute risk reduction, number needed to treat and tolerability. For clinicians, absolute benefit is often more important than relative hazard ratio. A 13-20% relative reduction in MACE may be transformative in very-high-risk secondary prevention but less compelling in low-risk primary prevention where the trial evidence is also weaker (4, 5, 23).

Subgroup findings should be interpreted with statistical restraint. Most CVOTs show broadly consistent relative effects across sex, age, baseline HbA1c, kidney function and BMI categories, but individual subgroup interactions are rarely powered to define treatment selection (4, 5, 27). Signals suggesting larger benefit at higher BMI, older age, greater inflammatory burden or established heart failure are hypothesis-generating unless prospectively confirmed (27). The absence of a significant subgroup interaction should not be mistaken for proof of identical benefit; it usually reflects limited power to detect heterogeneity.

### 9.2 Atrial fibrillation, stroke and arrhythmia biology

Stroke reduction has been a recurring signal in several GLP-1 RA analyses, particularly with semaglutide in SUSTAIN-6 and favourable stroke trends across pooled datasets (2, 5). Potential mechanisms include blood-pressure reduction, weight loss, endothelial improvement, anti-inflammatory effects, platelet modulation and reduced atrial substrate through epicardial fat reduction (19, 21, 43, 46). Because stroke is a heterogeneous endpoint, future trials should distinguish atherothrombotic, cardioembolic, lacunar and haemorrhagic stroke when possible.

Atrial fibrillation (AF) is an emerging but not yet definitive indication area. Obesity, epicardial adiposity, sleep apnoea, systemic inflammation, left atrial enlargement and HFpEF are shared substrates for AF (46, 47). GLP-1 RA-induced weight loss and inflammation reduction could plausibly reduce incident AF or improve rhythm-control outcomes after ablation (46). Meta-analyses and observational datasets have reported favourable AF signals, especially for semaglutide, but AF ascertainment has often been heterogeneous and not prospectively adjudicated as a primary endpoint (46). Dedicated randomized trials with continuous or systematic rhythm monitoring are required before GLP-1 RAs can be recommended specifically for AF prevention. AF should currently be considered exploratory and hypothesis-generating in the evidence hierarchy shown in **Figure 2**.

The small increase in resting heart rate associated with GLP-1 RAs complicates arrhythmia interpretation. A modest chronotropic effect does not necessarily translate into adverse outcomes, but it should prompt careful monitoring in patients with symptomatic tachyarrhythmias, advanced autonomic neuropathy, inappropriate sinus tachycardia or poorly controlled AF. For most patients, the net cardiometabolic effect appears favourable, but individualized monitoring remains prudent.

### 9.3 Special populations: older adults, women and Asian patients

Older adults represent a rapidly expanding target population because ASCVD, HFpEF, CKD and obesity frequently coexist in late life (38, 40). However, older patients also carry higher risks of sarcopenia, frailty, polypharmacy, dehydration, orthostatic hypotension and treatment discontinuation. Trial populations include older adults, but the frailest patients are often under-represented. In clinical practice, chronological age alone should not preclude therapy, but treatment goals should be explicit: preventing recurrent events, improving symptoms, preserving independence and avoiding functional decline. Weight loss should be monitored alongside strength, gait speed, nutritional intake and fall risk (48, 49).

Women are highly relevant to incretin-based cardiovascular medicine because obesity-related HFpEF disproportionately affects women and because weight-related stigma can influence both access and adherence (8, 40, 41). Yet women have historically been under-represented in cardiovascular trials relative to disease burden. Future analyses should report sex-specific absolute risk, tolerability, dose escalation, discontinuation and patient-reported outcomes. Pregnancy considerations are also important: incretin-based anti-obesity therapy is not used during pregnancy, and reproductive-age women require counselling regarding contraception and treatment discontinuation before planned pregnancy according to product labelling.

Asian populations require particular attention. Cardiometabolic risk frequently occurs at lower BMI thresholds in East and South Asian populations because visceral adiposity and beta-cell dysfunction may be more prominent at comparatively lower body weight (64, 65). A Western BMI threshold may therefore under-identify high-risk Asian patients who could benefit from cardiometabolic intervention (64, 65). Conversely, lower baseline body weight may increase the clinical relevance of lean mass loss during high-efficacy therapy. Trials and guidelines should therefore avoid assuming that identical BMI cutoffs capture equivalent risk across ethnic groups. Subgroup analyses should incorporate waist circumference, visceral adiposity, diabetes duration, kidney phenotype and body-composition endpoints rather than BMI alone.

### 9.4 Health economics, persistence and the translation gap

The gap between randomized-trial efficacy and real-world population benefit is likely to be large unless access and persistence improve (62, 63). GLP-1 RA therapy is expensive in many health systems, coverage criteria vary widely, and shortages have repeatedly disrupted continuity (63). From a cardiovascular prevention perspective, intermittent therapy is problematic because discontinuation often leads to weight regain and deterioration of risk factors (49). A drug with impressive relative efficacy can have limited public-health impact if only a minority of eligible patients start it and even fewer persist long term. Major implementation barriers are summarized in **Figure 4C**.

Cost-effectiveness depends strongly on drug price, baseline cardiovascular risk, duration of treatment, durability of weight loss, adverse-event rates and assumptions about long-term event reduction (63). Secondary-prevention populations with established CVD and obesity are more likely to meet cost-effectiveness thresholds than low-risk primary-prevention populations (6, 30, 63). Health systems should therefore prioritize patients with the highest absolute risk and the strongest outcome evidence while expanding manufacturing, price negotiation and equitable distribution. Coverage policies that restrict treatment only to extreme BMI categories may miss patients with established CVD, CKD or HFpEF who have substantial cardiometabolic risk at lower BMI (6–8, 64).

Persistence is a clinical skill as much as a patient behaviour (45, 49, 58). Clinicians should set expectations before initiation: nausea is common during escalation; maximum benefit develops over months; chronic therapy is usually required; and resistance exercise and nutrition are part of the prescription. Follow-up at 4–8 weeks can rescue many patients from discontinuation by adjusting titration, managing constipation, reviewing meal patterns and reframing therapy around cardiovascular protection rather than cosmetic weight loss. Multidisciplinary programmes that include nursing, dietetics, pharmacists and exercise professionals are likely to outperform isolated prescribing.

### 9.5 Phenotype-based clinical algorithm

A practical algorithm begins with phenotype rather than HbA1c (54–56). Step 1 is to identify the dominant high-risk phenotype: established ASCVD, obesity with established CVD, CKD with albuminuria, obesity-related HFpEF, symptomatic PAD or overlapping multimorbidity. Step 2 is to select an agent with direct outcome evidence for that phenotype when available (6–9). Step 3 is to layer complementary therapies - statins, antiplatelet or anticoagulant therapy when indicated, renin-angiotensin system blockade, SGLT2 inhibitors, finerenone, antihypertensive therapy, smoking cessation and exercise rehabilitation - rather than allowing one novel therapy to displace established standards (35–37, 57). This complementary-care strategy is summarized in **Figure 5C**, emphasizing that incretin therapy should be integrated with, rather than replace, guideline-directed cardiometabolic and vascular care. Step 4 is to personalize dose escalation, safety monitoring and persistence support.

For ASCVD plus T2DM, an outcome-proven GLP-1 RA is prioritized for atherosclerotic risk reduction, with SGLT2 inhibitor co-therapy when CKD or heart failure risk is present (1–3, 54, 55). For established CVD plus obesity without diabetes, semaglutide 2.4 mg is the outcome-proven option (6, 30). For CKD plus T2DM, semaglutide may be layered with SGLT2 inhibition and finerenone when indicated (7, 35, 37). For obesity-related HFpEF, semaglutide is used primarily for symptoms, function and weight, while SGLT2 inhibitors and careful diuretic management remain central (8, 41). For PAD, semaglutide should be positioned as a functional and cardiometabolic adjunct to comprehensive vascular care (9, 57).

This algorithm should be revised as new CVOTs report (24–26, 52). In particular, positive cardiovascular outcomes with tirzepatide or oral non-peptide GLP-1 RAs in non-diabetic CVD populations would alter sequencing, while neutral active-comparator results would support a more individualized choice based on weight, tolerability, route, cost and access rather than assumed cardiovascular superiority.

### 9.6 Regulatory nuance and labelling discipline

Regulatory approval should be described with the same precision as trial evidence. Semaglutide 2.4 mg has an FDA indication for reducing MACE risk in adults with established cardiovascular disease and overweight or obesity, but that indication does not automatically extend to lower doses, other GLP-1 RAs, dual agonists or oral small molecules (30). Conversely, approval of an anti-obesity medication without a cardiovascular indication should not be presented as cardiovascular proof (26). This distinction has practical implications for payer coverage, prescribing responsibility, patient consent, and scientific interpretation.

The rapid approval of oral non-peptide therapy also illustrates a new regulatory landscape (26). Delivery convenience may improve uptake, but expedited approval based on weight-management trials should be separated from cardiovascular outcome evidence (25, 26). Manuscripts in this field should therefore include an as-of date for regulatory statements, because labelling, shortages, indications and postmarketing safety requirements change rapidly. For a submission prepared in 2026, an explicit statement such as ‘regulatory status is described as of 30 April 2026’ prevents ambiguity during peer review and revision.

A similar discipline applies to guideline statements (35, 54, 55, 57). Guidelines frequently lag behind trial publication and vary between regions. A therapy may be evidence-supported before it is guideline-endorsed, and guideline-endorsed before it is accessible or reimbursed. Therefore, clinical recommendations should distinguish evidence, regulatory approval, guideline class, reimbursement and practical availability.

### 9.7 Standards for future mechanistic and translational studies

Mechanistic studies should move from isolated biomarker changes toward causal human biology. Ideal trial designs would embed vascular imaging, coronary plaque characterization, endothelial function testing, inflammatory proteomics, platelet function assays, kidney biomarker panels, body-composition measurement and tissue biobanking within randomized clinical trials (19, 21, 43). Serial measurements should be aligned with clinical event adjudication, because early biomarker changes and late event reduction may reflect different biological phases.

Weight-independent mechanisms require particularly careful study design (21, 27). Comparing individuals who lose similar amounts of weight through different interventions may help separate weight loss from drug-specific biology. Mendelian randomization, mediation analysis and omics discovery can generate hypotheses, but randomized designs with active comparators remain essential (27, 47). For example, comparing GLP-1 RA therapy with lifestyle- or surgery-induced matched weight loss could help determine whether incretin signalling has vascular effects beyond adiposity reduction.

Translational endpoints should also be clinically relevant. A reduction in hsCRP or epicardial fat is informative, but it becomes more persuasive when linked to improved endothelial function, lower plaque inflammation, better diastolic reserve, improved walking capacity or fewer adjudicated events (19, 21, 22, 46, 47). The next generation of mechanistic work should therefore be multidimensional rather than narrowly biomarker-driven.

## 10 Limitations of the evidence base

Several limitations constrain interpretation. First, not all incretin agents have the same cardiovascular evidence (5, 12, 13). Outcome-proven conclusions should be anchored to specific molecules and doses. Second, cross-trial comparisons are vulnerable to confounding by era, background therapy, baseline risk, endpoint definitions and adherence (4, 5, 23). Third, many mechanistic claims rely on animal models, cellular systems or biomarkers rather than human tissue-level causal evidence (19, 43). Fourth, mediation analyses can identify unexplained variation but cannot precisely assign a percentage of benefit to direct cardioprotection because unmeasured mediators, measurement error and time-varying confounding remain unavoidable (27).

Fifth, HFpEF and PAD trials to date emphasize patient-centred functional endpoints more than hard clinical events (8, 9, 41). These endpoints are clinically meaningful, but they should not be conflated with mortality or hospitalization evidence. Sixth, newer multi-agonists have impressive metabolic efficacy but limited completed CVOT evidence (24, 25, 52, 53). Seventh, long-term safety beyond trial duration, especially regarding sarcopenia, nutritional status, treatment interruption and effects in frail older adults, remains incompletely characterized (48, 49, 58). Finally, access and persistence are not peripheral implementation details; they determine whether trial-level efficacy becomes population-level benefit (62, 63).

## 11 Future directions

### 11.1 Precision medicine and responder identification

The next stage of incretin cardiovascular medicine should move beyond broad eligibility criteria toward responder phenotyping. Potential predictors include baseline ASCVD phenotype, obesity class, visceral adiposity, hsCRP, albuminuria, natriuretic peptides, hepatic steatosis, frailty, sex, ethnicity, gut microbiome composition and genetic variation in incretin-related pathways (27, 47, 64, 65). Machine-learning approaches may help identify response clusters, but they must be externally validated and clinically interpretable. The goal should not be to restrict therapy unnecessarily, but to match drug intensity, combination therapy and monitoring to the patients most likely to derive absolute benefit.

Biomarker-guided trials are particularly needed to determine whether anti-inflammatory response predicts MACE reduction, whether epicardial fat reduction predicts HFpEF improvement, and whether albuminuria reduction mediates kidney protection (21, 22, 27). These studies should include serial imaging, proteomics, metabolomics, body composition and adjudicated outcomes. Key translational opportunities—including weight-independent mechanisms, head-to-head cardiovascular trials, combination therapy, biomarker-guided selection, and oral delivery strategies—are summarized in **Figure 4B**.

### 11.2 Combination therapy trials

Layered cardiometabolic therapy is biologically rational, but dedicated combination trials remain limited (36, 37). SGLT2 inhibitors, GLP-1 RAs and finerenone affect partly distinct pathways: renal tubular energetics and haemodynamics, inflammation and adiposity, and mineralocorticoid-driven fibrosis and inflammation (36, 37). Trials should compare simultaneous versus sequential initiation, define safety monitoring for volume depletion and renal function, and evaluate patient-centred outcomes such as treatment burden, adherence and quality of life.

Combination therapy should also be assessed in patients without diabetes but with obesity, CKD, HFpEF or ASCVD (6, 7, 36, 37). The SELECT era makes it increasingly artificial to restrict cardiometabolic drug development to diabetes populations when the relevant biology includes adipose tissue, inflammation, kidney disease and vascular risk (6).

### 11.3 Cardiovascular outcomes for next-generation agents

Future CVOTs for tirzepatide, retatrutide, CagriSema and oral non-peptide GLP-1 RAs should not simply replicate older designs (24–26, 52, 53). They should include non-diabetic obesity with established CVD, HFpEF with obesity, CKD phenotypes, PAD populations, arrhythmia surveillance and body-composition substudies. Active-comparator designs will be increasingly important because placebo-controlled trials may become ethically and clinically less informative when outcome-proven therapy exists (3, 24).

The field should also define clinically acceptable trade-offs. A drug that produces greater weight loss but more discontinuation, greater lean mass loss or no incremental MACE reduction may be preferable for some patients and inferior for others (24, 48, 49, 52, 58). Cardiovascular benefit should be evaluated as net clinical benefit, incorporating events, symptoms, function, safety, treatment burden and access.

## 12 Conclusions

Incretin-based therapy has become one of the most important developments in contemporary cardiometabolic medicine. Long-acting GLP-1 RAs reduce atherosclerotic events in patients with T2DM and high cardiovascular risk, semaglutide 2.4 mg reduces MACE in established CVD with overweight or obesity without diabetes, and dedicated studies support clinically meaningful benefits in CKD, obesity-related HFpEF and symptomatic PAD. These findings justify a conceptual shift from diabetes adjunct therapy to evidence-based cardiovascular risk modification in carefully defined populations.

The strongest version of this conclusion is also the most disciplined: cardiovascular benefit is molecule-, dose-, population- and endpoint-specific. Semaglutide outcome evidence should not be generalized to all incretin-based drugs; multi-agonist metabolic potency should not be equated with cardiovascular outcome superiority; and mediation analyses should not be overread as precise quantification of direct cardioprotection. The future of the field depends on rigorous CVOTs, mechanistic human studies, combination therapy trials, body-composition and frailty research, and implementation science that addresses cost, persistence and equity.

If these priorities are met, incretin-based therapy will not merely add another drug class to cardiovascular prevention. It will redefine how cardiovascular medicine approaches obesity, inflammation, kidney disease, metabolic dysfunction and patient-centred functional limitation as integrated therapeutic targets.

## Glossary

ACS: acute coronary syndrome
ADA: American Diabetes Association
AF: atrial fibrillation
ASCVD: atherosclerotic cardiovascular disease
BMI: body mass index
CKD: chronic kidney disease
CVOT: cardiovascular outcome trial
CVD: cardiovascular disease
eGFR: estimated glomerular filtration rate
FDA: U.S. Food and Drug Administration
GIP: glucose-dependent insulinotropic polypeptide
GLP-1: glucagon-like peptide-1
GLP-1 RA: GLP-1 receptor agonist
HbA1c: glycated haemoglobin
HFpEF: heart failure with preserved ejection fraction
HR: hazard ratio
hsCRP: high-sensitivity C-reactive protein
KCCQ-CSS: Kansas City Cardiomyopathy Questionnaire Clinical Summary Score
MACE: major adverse cardiovascular events
MEN2: multiple endocrine neoplasia type 2
MTC: medullary thyroid carcinoma
PAD: peripheral artery disease
RAS: renin-angiotensin system
RCT: randomized controlled trial
SGLT2: sodium-glucose cotransporter-2
T2DM: type 2 diabetes mellitus
UACR: urine albumin-to-creatinine ratio
WHO: World Health Organization
